# Modulation of Interhemispheric Functional Coordination in Breast Cancer Patients Receiving Chemotherapy

**DOI:** 10.3389/fpsyg.2020.01689

**Published:** 2020-07-29

**Authors:** Longxiang Tao, Lu Wang, Xingui Chen, Fujun Liu, Feiyan Ruan, Jingjie Zhang, Li Shen, Yongqiang Yu

**Affiliations:** ^1^Department of Radiology, The First Affiliated Hospital of Anhui Medical University, Hefei, China; ^2^Department of Neurology, The First Affiliated Hospital of Anhui Medical University, Hefei, China; ^3^Anhui Province Key Laboratory of Cognition and Neuropsychiatric Disorders, Hefei, China; ^4^Department of Breast Surgery, The First Affiliated Hospital of Anhui Medical University, Hefei, China; ^5^Department of Radiotherapy, The First Affiliated Hospital of Anhui Medical University, Hefei, China

**Keywords:** breast cancer, chemotherapy, voxel-mirrored homotopic connectivity, resting-state functional connectivity, VMHC

## Abstract

**Objectives:**

Chemotherapy induces cognitive impairments including memory impairment attention deficit and executive dysfunction in patients with breast cancer (BC) during or after chemotherapy. Previous studies identified brain structural and functional changes in BC patients receiving chemotherapy; however, there are no studies assessing functional connectivity (FC) between homotopic brain regions in BC patients using a voxel-mirrored homotopic connectivity (VMHC) method. In the present study, we explored cognitive function and whole brain homotopic FC in BC patients receiving chemotherapy compared with healthy controls using the VMHC method.

**Methods:**

The present cross-sectional study included 35 premenopausal women with breast cancer who received chemotherapy, as well as 32 age- and sex-matched healthy controls (HC). All subjects underwent resting-state functional magnetic resonance imaging, which measured homotopic brain FC, and cognitive neuropsychological assessments evaluating attention, memory, and executive function domains.

**Results:**

The BC group had lower VMHC than the HC group in the cingulated posterior, insular and postcentral regions. No region exhibited higher VMHC in the BC group than in HC group. Correlation analysis in the BC group indicated that VMHC values in the cingulated posterior were significantly correlated with executive function tests, and that the VMHC values in the insular were significantly correlated with memory tests.

**Conclusion:**

The present study showed that VMHC decreased in different brain regions including cingulated posterior, insular and postcentral regions. A significant correlation was observed between the VMHC values in the brain regions and neuropsychological tests. These results suggested that changes in VMHC values in different brain regions may underlie cognitive changes in BC patients receiving chemotherapy.

## Introduction

Breast cancer (BC) is a common invasive disease in women. Surgery, radiotherapy, and chemotherapy have improved the survival of patients with BC. There is evidence that chemotherapy induces cognitive impairments including memory impairment, attention deficit, and executive dysfunction in patients with BC (Tong et al., 2018). These cognitive impairments affect long-term quality of life.

Previous neurophysiological studies reported that BC patients show obvious subjective and objective cognitive changes after completing chemotherapy (Ahles et al., 2010; Janelsins et al., 2017). A meta-analysis showed that cognitive impairments in BC patients mainly involved reduced attention, memory, and executive function (Jim et al., 2012). However, the data from the International Cognition and Cancer Task Force (ICCTF) show cognitive impairments in 13–70% of patients after chemotherapy treatment (Wefel et al., 2011). Previous studies have shown that the magnitude of cognitive impairment in BC survivors showed small to moderate effect sizes (Falleti et al., 2005; Jim et al., 2012). Moreover, one cognitive function study suggested that the decrease in attention in patients is not associated with chemotherapy. These inconsistent results may be a result from differences in experimental design and population heterogeneity (Vardy et al., 2007; Barton, 2013). Therefore, it is essential to select a consistent group of neurophysiological tests to assess cognitive impairment.

Advances in neuroimaging technology have enabled researchers to investigate the effects of chemotherapy treatment on brain structure and function and to understand the neural mechanisms associated with BC. Previous studies showed that BC patients have brain structure changes after adjuvant chemotherapy, such as reduced gray matter and alterations in white matter. In addition, brain functional changes were observed in BC patients, such as decreased efficiency of functional connectivity (FC) and neural networks in a resting state (Piccirillo et al., 2015; Tao et al., 2017), and cerebral activation alterations during the performance of cognitive tasks (Silverman et al., 2007). Recently, researchers used resting-state FC to explore the neural activity of brain regions (Fox and Raichle, 2007; Li et al., 2019; Liao et al., 2019). Our previous study showed that BC patients receiving chemotherapy have abnormal FC of the posterior cingulate cortex. Although this finding supports the dysconnectivity hypothesis and the presence of interhemispheric interaction deficits in BC patients receiving chemotherapy, there are currently no studies assessing FC among homotopic brain regions in BC patients using a voxel-mirrored homotopic connectivity (VMHC) method. VMHC is a validated method to assess the resting-state FC between each voxel in one hemisphere and its mirrored voxel in the opposite hemisphere (Zuo et al., 2010; Lysaker et al., 2019). This method is an effective and sensitive technique for evaluating interhemispheric functional coordination (Wei et al., 2014; Shi et al., 2018) and has been successfully applied in cognitive neuroscience studies and clinical studies (Hoptman et al., 2012; Molina et al., 2013; Zhou et al., 2013).

In the present study, we explored cognitive function and whole-brain homotopic FC in BC patients after chemotherapy compared with healthy controls (HCs) using a VMHC method. Based on the findings of previous studies, we hypothesized that patents with BC would have cognitive impairments and reduced VMHC in specific brain regions. In addition, we examined the potential relationships between cognitive impairments and VMHC changes.

## Materials and Methods

### Participants

We recruited 35 BC patients from the Department of Breast Surgery, the First Affiliated Hospital of Anhui Medical University, and these patients regularly received adjuvant chemotherapy for 6 weeks. All patients were stages I–III and ranged in age from 28 to 52 years. At the same time, we also recruited matched healthy people from the surrounding communities. The inclusion criteria were as follows: (1) postoperative pathology suggested new BC; (2) adopt standard doses of adjuvant chemotherapy drugs (without hormones) mainly including doxorubicin, paclitaxel, and cyclophosphamide; and (3) Beijing version of Montreal Cognitive Assessment (MoCA) test score ≥24 points (Liu et al., 2019). Exclusion criteria are as follows: (1) receiving endocrine therapy; (2) distant metastasis or cachexia; (3) acute side effects such as weakness, anemia, nausea, vomiting after chemotherapy; (4) traumatic brain injury, neuropsychiatric disease, claustrophobia, other underlying diseases that do not allow MRI scans. See Table 1 for details.

**TABLE 1:** Demographic characteristics of patients and healthy controls recruited for this study.

	**HC group (*n* = 32)**	**BC group (*n* = 35)**		
	**Mean or count (SD)**	**Mean or count (SD)**	***t***	***p***
Age (years)	41.12 (7.42)	41.66 (6.29)	−0.317	0.752
Education (years)	10.16 (2.16)	11.11 (2.77)	−1.568	0.122
**Breast cancer stage**				
I	NA	2	NA	NA
II	NA	22	NA	NA
III	NA	11	NA	NA
Received radiotherapy	NA	29	NA	NA
Days after adjuvant chemotherapy	NA	25.06(5.04)	NA	NA
HAMA	4.47(1.08)	4.86(1.29)	−1.333	0.187
HAMD	4.69 (1.09)	4.94 (1.11)	−0.948	0.346
Fatigue	19.62 (3.89)	23.29 (2.41)	−4.580	<0.001
MoCA	26.44 (1.13)	25.89 (1.29)	1.862	0.067

*HC, healthy control; BC, breast cancer; SD, standard deviation; NA, not applicable; HAMA, Hamilton Anxiety Rating Scale; HAMD, Hamilton Depression Rating Scale; MoCA, Montreal Cognitive Assessment Test.*

None of the HCs had a history of alcohol or drug abuse or neurological and psychiatric disorders. Participants did not have subtle or severe affective disorders [Hamilton Depression Rating Scale (HAMD) scores ≤7 and/or Hamilton Anxiety Rating Scale (HAMA) scores ≤7]. For both patients and HCs, all participants were right-handed, could understand the study procedures and speak Chinese, and had corrected-to-normal vision. The current study was approved by the Research Ethics Committee of the First Affiliated Hospital of Anhui Medical University, China. All participants or their guardians provided informed, written consent.

### Neuropsychological Background Tests

All participants were assessed using a neuropsychological background test to investigate cognitive function and affective disorders during daily life. In general, the MoCA test was used to assess global normal cognitive function. Digit Span (DS) was assessed to measure attention function. The Stroop Color Word Test and Trail Making Test (TMT) were used to assess executive function performance. The Chinese version of the Auditory Verbal Learning Test (AVLT) was used to investigate memory function. The China Version of the Cancer Related Fatigue (CRF) was used to exclude possible acute fatigue symptoms (inclusion criterion: <28 points). The HAMD and HAMA tests were administered to assess the participants’ depression and anxiety symptoms, respectively. All neuropsychological tests were performed by skilled psychologists. Completion of these tests took approximately 90 min for each participant.

### MRI Data Acquisition

In order to minimize head movements, patients were instructed to use comfortable foam padding before scanning, and earplugs were inserted during scanning to reduce the noise of the scanner. The T1-weighted three-dimensional spoiled gradient recalled acquisition in steady state (3D-SPGR) images were obtained with the following parameter settings: repetition time (TR) = 7.2 ms; echo time (TE) = 3.1 ms; flip angle = 12°; slice thickness = 1 mm; field of view (FOV) = 256 mm^2^ × 256 mm^2^; matrix size = 256 × 256; and 172 sagittal slices. Subsequently, functional images were acquired using a gradient-recalled echo planar imaging sequence (TR = 2,000 ms, TE = 35 ms, and flip angle = 90°). Thirty transverse slices (FOV = 240 mm^2^ × 240 mm^2^, matrix = 64 × 64, slice thickness = 3.6 mm, inter-slice gap = 0.4 mm, and 240 volumes) aligned along the anterior commissure–posterior commissure line were acquired. During the rs-fMRI scans, all subjects were instructed to keep their eyes closed, relax and move as little as possible, think of nothing in particular, and not fall asleep. All participants collected MRI data for two modalities.

### Data Processing

Functional image processing was performed using the DPARSF^1^ and SPM8^2^ toolkits. The specific image preprocessing process includes DICOM format conversion, removal of the first 10 time points, interlayer time correction, head movement correction, data removal with translation of head movement >2 mm and/or rotation >2°, and high-resolution T1. The structural images are registered to the standard Montreal space, and the DARTEL segmentation algorithm is used to separate white matter, gray matter, and cerebrospinal fluid (spatial resolution: 3 × 3 × 3) on the T1 structure image. The image is registered to the standard Montreal space and resampled to 3 mm isotropic voxels. Finally, the image data are spatially smoothed, filtered (0.01–0.08 Hz), and de-linearized to reduce low-frequency drift and high-frequency physiological noise. Finally, covariates were removed using linear regression (head movement, whole brain, cerebrospinal fluid, white matter signal). VMHC is also performed through the DPABI toolkit, extracting subjects preprocessed (all the normalized gray matter images were averaged to create a mean image, then the generated mean image was then averaged with its left–right mirrored version to obtain a symmetrical template and mask for VMHC) and registered to each voxel time series in the standard Montreal space hemisphere and calculating the Pearson correlation coefficient (VMHC value) between it and the contralateral mirror voxel. Fisher z-transforms were used to improve the normal distribution, and the results were used for analysis between VMHC groups.

### Statistical Analysis

Clinical and demographic data were analyzed using SPSS 19.0 (SPSS, Chicago, IL, United States). The unpaired two-sample *t*-test was used to evaluate changes of VMHC between two groups using a toolbox in SPM12, Statistic non-Parameter Mapping (SnPM). To control the family-wise error in multiple comparisons, we first set a cluster-defined threshold *t* of 2.39 (corresponding to *p* = 0.01 at the voxel level). Then, only clusters larger than 30 volume were reported as cluster-level corrected, Pcorr < 0.05, within the unilateral hemisphere of the symmetric template. Pearson’s correlation analysis was used to explore the associations between the VMHC values in significant clusters and the score of neuropsychological background tests, with *p* < 0.05 set as the significance level. The metering data were presented as the mean ± SD.

## Results

### Neuropsychological Background Tests

The results of demographic and neuropsychological background tests are shown in Table 1. There were no significant differences in the demographic information, including age and the level of education, and neuropsychological performance on MoCA, DS, HAMA, and HAMD between the two groups (*p* > 0.05). The BC group showed significantly higher scores than the HC group in fatigue testing; however, all participants had scores that were below the cutoff value (CRF < 28 points).

The performance of the BC group in tests assessing memory (Delayed recall), as well as three subtests assessing executive function (the TMT B, Stroop Color Word Test, and Stroop Interference Test), was significantly worse than that of the HC group. Compared with the HC group, the BC group showed cognitive function deficits in various domains. The results of the neuropsychological assessment are shown in Table 2.

**TABLE 2:** Summary of neuropsychological background test.

	**HC group (*n* = 32)**	**BC group (*n* = 35)**	***t***	***p***
	**Mean (SD)**	**Mean (SD)**		
Attention/concentration				
WAIS Digit Span (forward)	5.87 (1.04)	5.91 (0.95)	−0.162	0.872
WAIS Digit Span (backward)	4.97 (0.90)	4.63 (1.03)	1.434	0.156
Stroop Color test (sec)	15.56 (3.27)	16.93 (3.88)	−1.559	0.124
Trail making A (sec)	53.59 (9.12)	54.02 (9.90)	−0.188	0.852
Memory (AVLT)				
Immediate Recall	10.72 (1.73)	10.86 (1.59)	−0.341	0.734
Delayed Recall	9.72 (1.82)	8.77(1.66)	2.227	0.029
Recognition	8.69 (2.05)	7.74 (1.96)	1.918	0.060
Executive function				
Trail making B (sec)	96.12 (13.73)	106.71 (11.99)	−3.370	0.001
Stroop Word test (sec)	18.78 (2.99)	21.05 (4.70)	−2.376	0.021
Stroop Interference test (sec)	31.56 (5.80)	36.04 (7.87)	−2.631	0.011

*HC, healthy control; BC, breast cancer; SD, standard deviation; AVLT, Auditory Verbal Learning Test.*

### Voxel-Mirrored Homotopic Connectivity: Group Differences

**FIGURE 1:**
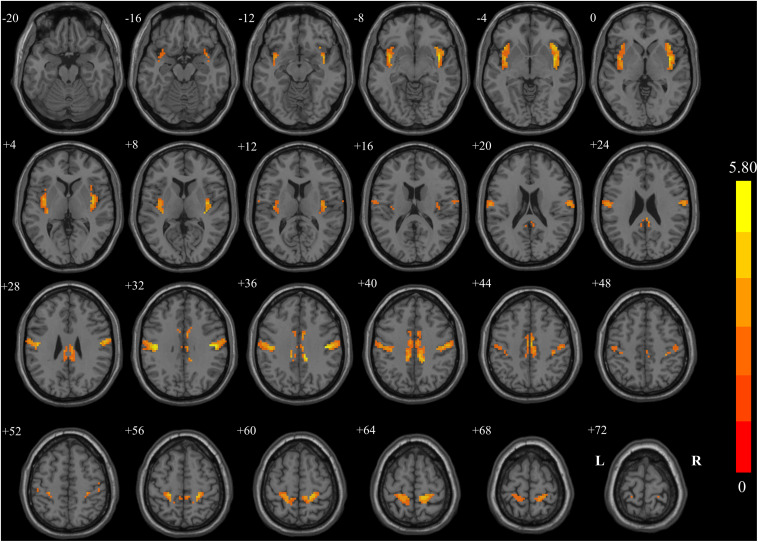
Regions showing significant changes in voxel-mirrored homotopic connectivity (VMHC) between breast cancer (BC) patients and healthy controls (HCs) with two-dimensional structural drawing. Statistical maps of voxel *t*-values of VMHC between BC and HC. The left side of each image corresponds to the left hemisphere of the brain, yellow denotes higher VMHC values (BC < HC), and the color bar indicates T values from *t*-tests between groups.

Figure 1 and Table 3 show the group comparisons of VMHC values. The BC group had lower VMHC than the HC group in the cingulated posterior, insular, and postcentral regions. No region exhibited higher VMHC in the BC group than in the control group.

**TABLE 3:** Brain regions with significant group effect in VMHC between the HC group and BC group.

**Brain region**	**MNI coordinate (peak voxel)**			**Brodmann area**	***p* (peak FEW corrected)**	**Peak *t* value**	**Cluster size**
	**X**	**Y**	**Z**				
**BC patients < healthy controls**							
Cingulate posterior	±12	−36	36	N	0.0024	5.75	319
Insular	±39	0	−6	48	0.0036	5.80	244
Postcentral	±48	−18	30	3	0.0062	5.15	183

*VHMC, voxel-mirrored homotopic connectivity; BC, breast cancer; HC, healthy control; MNI coordinates, Montreal Neurological Institute coordinates of the peak voxel; FEW, family-wise error; N, None; t, statistical value of the peak voxel; 1 voxel = 3 mm × 3 mm × 3 mm. VMHC computation bases on a symmetrical template, so the MNI coordinates of the peak voxel is also symmetrical.*

### Correlation Analysis of Neuropsychological Test Scores With Voxel-Mirrored Homotopic Connectivity

**FIGURE 2:**
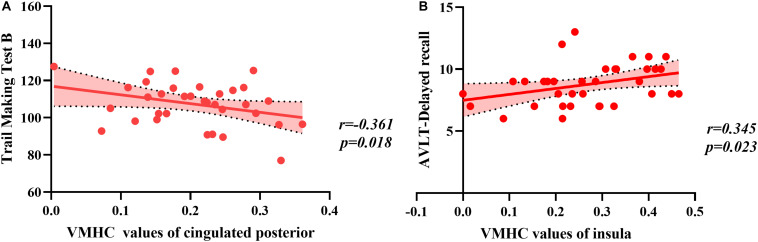
The correlation between regions and values of voxel-mirrored homotopic connectivity (VMHC) and neuropsychological scores. **(A)** The Trail Making Test B raw scores were negatively correlated with VMHC values of the cingulated posterior. **(B)** The Delayed recall raw scores were positively correlated with VMHC values of the insula.

There was a significant difference between the BC group and HC group in the CRF test. Because high fatigue scores may interfere with cognition in BC patients, we conducted partial correlations with fatigue as the factor. Correlation analysis in the BC group indicated that VMHC values in the cingulated posterior were significantly correlated with a measure of executive function (TMT B, *r* = −0.361, *p* = 0.018) and that the VMHC value in the insular was significantly correlated with assessing memory (Delayed recall, *r* = 0.345, *p* = 0.023) (Figure 2). No significant correlations were observed between VMHC values in the postcentral region and neuropsychological test scores (all *p* > 0.05) (as shown in Table 4). There were no significant correlations between neuropsychological test scores and VMHC values in the HC group (all *p* > 0.05).

**TABLE 4:** Correlations among the VMHC scores and neuropsychological tests (raw scores) for the BC group patients.

	**DS**		**TMT**		**Stroop Color Word Test**			**AVLT**		
	**Forward**	**Backward**	**A test**	**B test**	**Color**	**Word**	**Interference**	**Immediate**	**Delayed**	**Recognition**
Cingulate posterior	*r* = 0.153	*r* = 0.165	*r* = −0.030	*r* = −0.361	*r* = 0.055	*r* = −0.042	*r* = −0.238	*r* = −0.033	*r* = 0.038	*r* = 0.011
	*p* = 0.194	*p* = 0.176	*p* = 0.433	*p* = 0.018*	*p* = 0.378	*p* = 0.407	*p* = 0.088	*p* = 0.427	*p* = 0.415	*p* = 0.476
Insular	*r* = 0.068	*r* = 0.107	*r* = −0.164	*r* = 0.006	*r* = −0.065	*r* = 0.002	*r* = −0.171	*r* = 0.192	*r* = 0.345	*r* = 0.194
	*p* = 0.351	*p* = 0.273	*p* = 0.176	*p* = 0.486	*p* = 0.357	*p* = 0.495	*p* = 0.167	*p* = 0.138	*p* = 0.023*	*p* = 0.136
Postcentral	*r* = 0.037	*r* = 0.068	*r* = 0.087	*r* = −0.220	*r* = 0.057	*r* = −0.042	*r* = −0.267	*r* = −0.075	*r* = 0.272	*r* = −0.006
	*p* = 0.418	*p* = 0.351	*p* = 0.312	*p* = 0.105	*p* = 0.373	*p* = 0.407	*p* = 0.063	*p* = 0.337	*p* = 0.060	*p* = 0.487

VMHC, voxel-mirrored homotopic connectivity; BC, breast cancer; DS, Digit Span; TMT, Trail Making Test; AVLT, Auditory Verbal Learning Test. *p < *0.05*.

## Discussion

To the best of our knowledge, this is the first study to investigate interhemispheric coordination in BC patients treated with chemotherapy. The results showed that patients in the BC group had significant cognitive impairments in memory and executive function. In addition, we observed decreased VMHC in the cingulated posterior, insular, and postcentral regions. There were significant correlations between the executive function changes and VMHC values in the cingulated posterior, as well as the memory deficits and VMHC values in the insular.

Previous studies reported cognitive impairments in BC survivors specifically in the domains of immediate and delayed recall memory, attention, executive function, and processing speed (Janelsins et al., 2011; Edelstein and Bernstein, 2014). Analogously, we found that the BC group of patients had significant cognitive impairments in tests assessing delayed recall memory and executive function in the present study. However, these findings were not consistent with results of previous studies (Edelstein and Bernstein, 2014), which could be attributed to intraindividual variability and differences in evaluation criteria.

To investigate the mechanism underlying cognitive impairment, we used the functional MRI technique to analyze VMHC in patients with BC receiving chemotherapy. Our results showed that BC patients had decreased VMHC in the insular, cingulated posterior, and postcentral regions. These findings were consistent with those of previous neuroimaging studies that identified structural and functional changes associated with chemotherapy, including changes in gray and white matter (Deprez et al., 2012) and cerebral activation during the performance of cognitive tasks (Reuter-Lorenz and Cimprich, 2013; Deprez et al., 2014). These brain changes also included the insular and cingulated structures and functional changes. Previous neuropsychological and neuroimaging studies suggested a close association between the changed brain regions observed in the current study and cognitive function. A brain injury study showed that injury of the cingulate gyrus was associated with worse performance in memory, attention, and executive function tests (Baek et al., 2013; Yoo et al., 2014; Jang and Kwon, 2016). The results of diffusion tensor MRI verified the relationship between the cingulate gyrus and cognitive impairment (Koenig et al., 2015). In this study, we found a significant negative correlation between decreased VMHC in the cingulated posterior and the response times on the TMTs in BC patients. These results suggest that the patients with BC spent a longer time resolving the performance of executive function tests and that this deficit may result from the brain function changes which had decreased VMHC in the cingulated posterior.

Other studies reported that patients with insular lesions showed significantly worse performance in most neurocognitive domains including learning and memory tests (Wu et al., 2011; Terasawa et al., 2015). The brain network composed of the insula was also confirmed to affect memory and executive function (Christopher et al., 2014; Piccirillo et al., 2015). Consistent with these findings, the present results showed that BC patients had decreased VMHC in the insula, as well as a correlation between decreased VMHC and memory deficit. These findings suggested a potential neurological mechanism underlying chemotherapy-induced cognitive function impairment in BC patients.

The current study strongly suggested that cognitive impairment and decreased VMHC exist in BC patients receiving chemotherapy. However, the present study had several limitations. First, our study failed to recruit the control group with patients not receiving chemotherapy or before receiving chemotherapy. Therefore, the effect of the tumor on cognitive impairment could not be excluded. A disease control group needs to be added in future studies to rule out the effects of cancer on cognitive impairment. Second, only the VMHC value was used to explore the mechanism of cognitive impairment in this study. Combining structural and functional analyses would be an accurate method to examine the mechanism in future studies.

## Conclusion

In summary, we showed that VMHC decreased in different brain regions including the cingulated posterior, insular, and postcentral regions. We observed a significant correlation between the results of subtests assessing neuropsychological functions such as executive function and VMHC values in the cingulated posterior, as well as memory and VMHC values in the insular. These results suggested that changes in VMHC values in different brain regions may underlie the cognitive impairment in BC patients receiving chemotherapy.

## Data Availability Statement

The raw data supporting the conclusions of this article will be made available by the authors, without undue reservation.

## Ethics Statement

The studies involving human participants were reviewed and approved by the First Affiliated Hospital of Anhui Medical University, Hefei, China. Written informed consent to participate in this study was provided by the participants’ legal guardian/next of kin. Written informed consent was obtained from the individual(s), and minor(s)’ legal guardian/next of kin, for the publication of any potentially identifiable images or data included in this article.

## Author Contributions

LT, LW, LS, and YY designed the study and wrote this manuscript, which all authors have reviewed. LT, LW, XC, and FL acquired behavior and imaging data. FR and JZ recruited breast cancer patients. LT, LW, and XC analyzed the imaging data. All authors approved the final version to be published and certified that no other individuals not listed as authors have made substantial contributions to the manuscript.

## Conflict of Interest

The authors declare that the research was conducted in the absence of any commercial or financial relationships that could be construed as a potential conflict of interest.

## Footnotes

1 http://rfmri.org

2 www.fil.ion.ucl.ac.uk/spm
